# Adrenal neuroblastoma in an elderly adult: a case report and review of the literature

**DOI:** 10.1186/s13256-019-2204-7

**Published:** 2019-09-10

**Authors:** Haibin Zhang, Ziwei Feng

**Affiliations:** 1 Zhongshan School of Medicine, Sun Yat-sen University, Guangzhou, China; 2 Division of Urology, Guangdong General Hospital, Guangdong Academy of Medical Sciences, No.106 Zhongshan Er Road, Guangzhou, 510080 Guangdong China

**Keywords:** Neuroblastoma, Adrenal, Elderly, Adult

## Abstract

**Background:**

Neuroblastoma is an embryonal malignancy of the autonomic nervous system and is the most common extracranial tumor of early childhood. However, neuroblastoma in adults is rare with an overall incidence of 1 in 10 million adults/year. Adults with neuroblastoma have a significantly worse prognosis than children with neuroblastoma.

**Case presentation:**

In this case report we describe a 75-year-old Han Chinese woman with bilateral lower extremities weakness, numbness, and fatigue for 1 week. She initially presented as primary hyperaldosteronism with hypertension, persistent hypokalemia, and an elevated aldosterone/plasma renin activity ratio. An abdominal computed tomography scan with intravenously administered contrast showed a solid mass arising from her left adrenal gland. She underwent retroperitoneal laparoscopic surgery that allowed the resection of the mass. Microscopic and immunohistochemical staining, which were positive for synaptophysin, CD56, and vimentin, confirmed the diagnosis of adrenal neuroblastoma. Surgical resection of the tumor was done and no chemotherapy or radiation therapy was done postoperatively. She died from lung and brain metastases 22 months after surgical resection.

**Conclusion:**

Adrenal neuroblastoma in elderly adults is a very rare disease with sparse data available in the literature. Early stage disease could be managed by surgical resection alone. However, the prognosis is significantly worse than that observed in pediatric patients. Further research focusing on tumor biology and therapy for this rare malignancy in adults may help to improve disease outcome.

## Introduction

A neuroblastoma (NB) is an embryonal malignancy of the autonomic nervous system and is the most common extracranial cancer of early childhood and the most frequently diagnosed malignancy in infancy. Common locations where NB can occur include the adrenal medulla (at least 50%), neck, chest, and pelvis [1, 2]. The median age at diagnosis is 2 years, and more than 90% of patients are diagnosed under 10 years of age [3, 4]. The incidence of NB in adults is rare especially in the elderly population [5]. An adult with NB has a significantly worse prognosis than children, which may be due to different clinical course and response to therapy [3, 6]. We discuss a rare case of an elderly patient who presented with lower extremities weakness and hypokalemia and was diagnosed as having adrenal NB.

## Case presentation

**Fig. 1:**
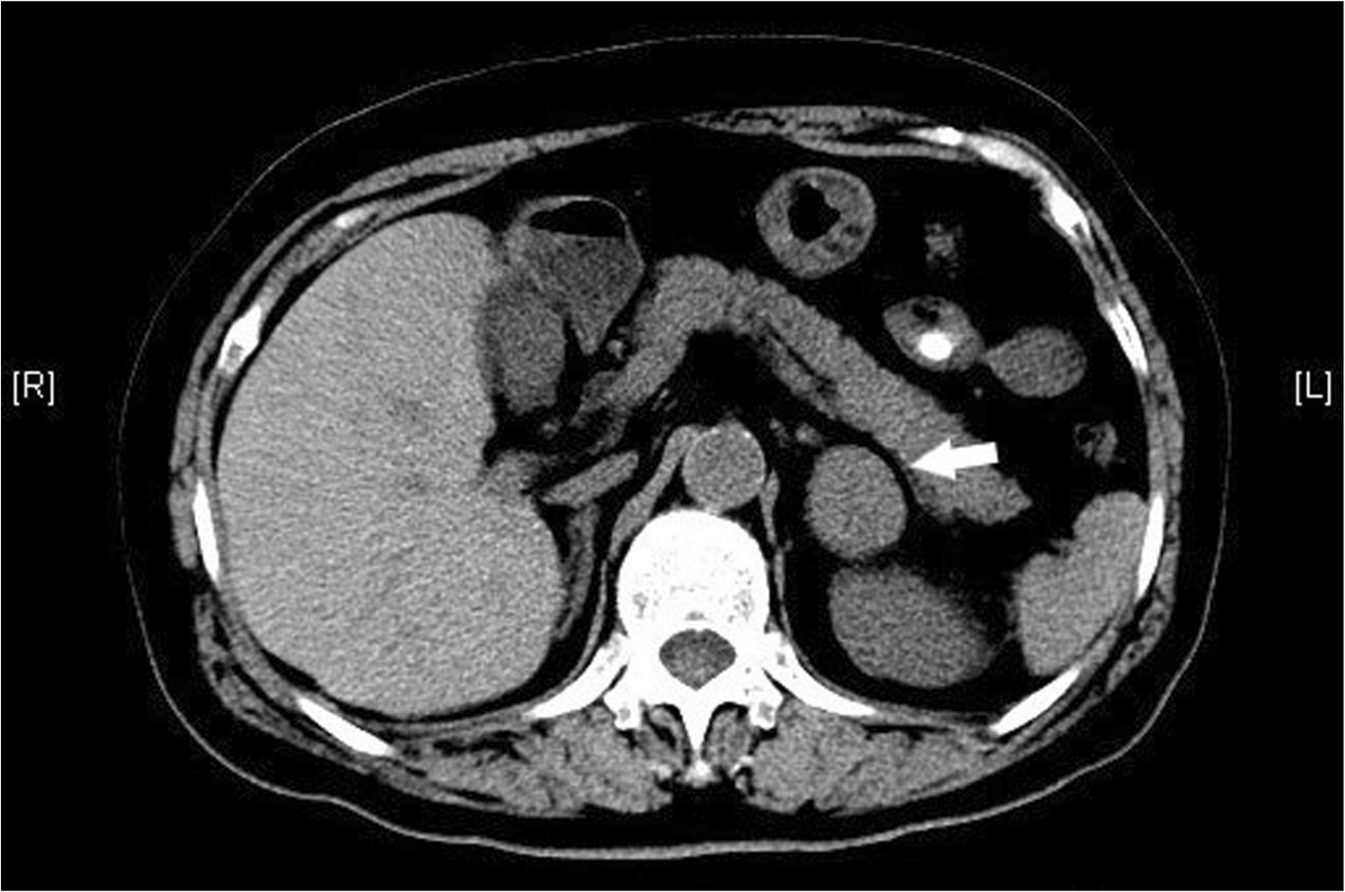
Abdominal computed tomography image of a 4.5 cm × 3.1 cm solid left adrenal mass (*arrow*)

**Fig. 2:**
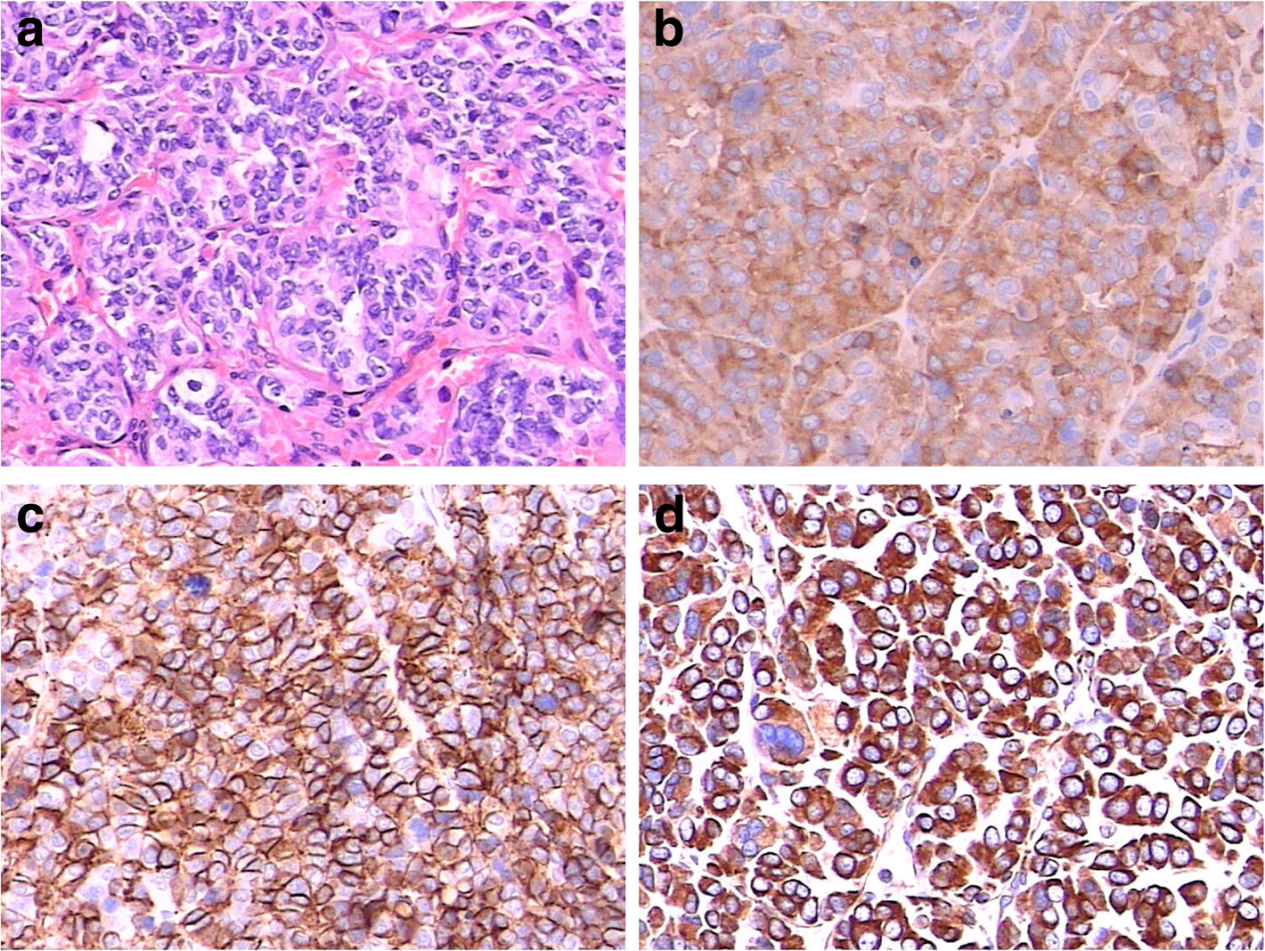
Photomicrographs revealed various tumor properties. **a** Microscopy revealed a tumor composed of small round blue cells forming rosettes with scant cytoplasm, fibrillary matrix material, hyperchromatic nuclei, and atypical mitoses. The tumor stained positively for **b** synaptophysin, **c** CD56, and **d** vimentin

A 75-year-old Han Chinese woman originally presented with bilateral lower extremities weakness, numbness with difficulty walking, and fatigue for 1 week. Her past medical history was unremarkable and she was healthy in general. She did not smoke tobacco or consume alcohol. She took no medication at home. She was married and had two children and both were healthy. There was no history of malignancies in her family. Her blood pressure was 147/100 mmHg, heart rate was regular 82/minute, and her body mass index (BMI) was 28.8 kg/m^2^. A physical examination was normal. A laboratory workup disclosed decreased serum potassium level of 2.7 mEq/L and a normal serum sodium level of 135.9 mEq/L. Her kidney function was within normal limits with blood urea nitrogen (BUN) of 8.9 mg/dL and a creatinine level of 0.79 mg/dL. Further testing was notable for an elevated serum aldosterone level of 174 ng/dL (normal ≤ 21 ng/dL), normal plasma renin activity (PRA) level of 7.59 ng/mL per hour, and elevated level of aldosterone/PRA of 22.9. Her 24-hour urine cortisol, vanillylmandelic acid (VMA), and metanephrine levels were unremarkable. She was initially diagnosed as having primary hyperaldosteronism and was given orally administered potassium and spironolactone. Muscle weakness and numbness were partially relieved. However, her serum potassium level was not corrected. Abdominal computed tomography (CT) scans with intravenously administered contrast showed a 4.5 cm × 3.1 cm solid mass arising from her left adrenal gland (Fig. 1). Subsequently, she underwent retroperitoneal laparoscopic surgery that allowed the resection of the mass originating in her left adrenal gland. All the solid mass was excised, whereas her left adrenal gland was carefully dissected and spared. The mass was well circumscribed and its cut surface was grayish brown. It was surrounded by normal adrenal tissue. On macroscopic examination, the grayish brown mass measured 5.6 × 4.5 × 2.5 cm. Microscopy revealed a tumor composed of small round blue cells forming rosettes with scant cytoplasm, fibrillary matrix material, hyperchromatic nuclei, and atypical mitoses (Fig. 2a). Immunohistochemical staining for CD99, epithelial membrane antigen, MyoD1, muscle actin monoclonal antibody (HHF35), chromogranin A, and S-100 was negative with strong positive staining for synaptophysin (Fig. 2b–d), CD56, vimentin, and Ki67 (+ 30%). On the basis of these findings, an adrenal NB was diagnosed. An I123-meta-iodobenzylguanidine (MIBG) scan was done postoperatively, which showed no hint of bone metastasis. No chemotherapy or radiation therapy was done based on our patient’s decision. However, she died 22 months after resection due to bilateral lung and brain metastasis.

## Discussion

NB is an embryonal tumor originating from the autonomic nervous system. The cell of origin is thought to be a developing and incompletely committed precursor cell derived from neural crest tissue. NB is the third most common childhood cancer [5]. However, the diagnosis is extremely rare in elderly patients with less than 100 cases reported in the literature. Our patient presented with NB in her adrenal gland associated with muscle weakness and persistent hypokalemia.

NB is rarely reported in the adult population, with < 10% of the cases diagnosed after age 10. The incidence rate for patients aged over 30 is 0.2 cases per million inhabitants per year and its incidence becomes increasingly scarce in the elderly population [5]. The clinical manifestation of NB in adult patients varies from asymptomatic to critically ill. It depends on the tumor’s primary location and the extent of metastatic disease. The most common primary sites in an adult patient (18–60 years) are central nervous system (39%) followed by retroperitoneum (17%), while in elderly patients (> 60 years) the most common primary sites are soft tissue including heart (60%) followed by trachea, mediastinum, and respiratory organs (31.4%) [1]. Therefore, the most common presentations are abdominal mass, abdominal pain, muscle weakness, or presentations due to metastases such as back pain, bone marrow failure, spinal compression, and hypertension [4, 5, 7]. Besides muscle weakness, our patient also presented with hyperaldosteronism which is quite rare and is considered an unexpected atypical presentation according to the medical literature [8]. The hyperaldosteronism can be explained by increased renin secretion secondary to the mass effect of the tumor compressing the renal artery [9].

A new International Neuroblastoma Risk Group (INRG) tumor staging system has been developed for the stratification of statistical and clinical risks of different subgroups of patients with NB. In the INRG Staging System, the extent of locoregional disease is determined by the absence or presence of image-defined risk factors (L1 and L2, respectively). Stage M is used for widely disseminated disease [10]. Four broad categories of very low risk, low risk, intermediate risk, and high risk were proposed in terms of 5-year event-free survival with rates of > 85%, > 75 to ≤ 85%, > 50 to ≤ 75%, and < 50%, respectively. The category is based on age at diagnosis, INRG tumor stage, histologic category, grade of tumor differentiation, DNA ploidy, and copy number status at the *MYCN* oncogene locus and at chromosome 11q [2]. Our patient was classified as stage L2 of the INRG Staging System [10].

In our case, regarding treatment, we only performed surgical resection of the tumor but no chemotherapy or radiation therapy was done after the operation. The treatment strategy in pediatric patients with NB has been well studied; it includes surgical resection, and optimal combination chemotherapy and radiotherapy. However, there are no standard treatment guidelines or chemotherapy protocols for adult or elderly patients with NB due to the rarity of NB in this population [5]. A cohort study yielded 118 adult patients with NB from University of Texas MD Anderson Cancer Center; it concluded that for adult patients with L1 and L2 disease, a combination of surgical resection and radiotherapy offered better progression-free survival as well as overall survival than surgical resection alone. The median progression-free survival in patients with L1 and L2 disease was 11.1 months and 5.9 months, respectively. Chemotherapy did not show any additional benefit in outcomes among patients with L1 and L2 disease. The most common chemotherapy regimens employed in adult patients with M stage disease were: cisplatin and etoposide alternating with carboplatin, vincristine, and cyclophosphamide (29%); and vincristine and cyclophosphamide alternating with cisplatin, doxorubicin, and dacarbazine (24%). The median overall survival of patients with M stage disease was 1.6 years [7]. Podda *et al.* conducted a series with 27 patients with NB aged 12–69 years in Italy [4]. The treatment protocol and outcome was as follows. Surgery only in stage I and surgery followed by radiotherapy in stage II. The 5-year overall survival rate was 83% for stage I and II disease. Chemotherapy consisted of six cycles of cisplatin and etoposide alternating with Adriamycin (doxorubicin), cyclophosphamide, and vincristine and local therapy after sixth course consisting either of radiotherapy or surgery in stage III. In stage IV, ifosfamide was added to the cycles applied in stage III, followed by a consolidation phase with 10 Gy fractionated hemibody irradiation (HBI) or autologous stem cell rescue, and local therapy with surgery or radiation was scheduled after the fourth cycle. The 5-year overall survival rate for patients with stage III and IV disease was 28% but all patients with stage IV disease relapsed and died due to disease progression [4].

## Conclusion

NB in elderly adults is a very rare disease with sparse data available in the literature regarding natural history, genetic causes, treatment, and outcomes. Early stage adrenal NB in elderly patients can be managed with surgical resection alone. However, elderly adult patients have a worse prognosis than is observed in pediatric patients. Here we reported a rare case of an elderly patient with adrenal NB treated with surgical resection and with a survival of 22 months. According to the medical literature, surgical resection combined with local radiation therapy offers better outcomes in cases of adult local NB. Chemotherapy should be considered in disseminated disease. Further research focusing on tumor biology and therapy for this rare malignancy in adults may help to improve disease outcome.

## Data Availability

Not applicable.

## Abbreviations

BMI: Body mass index
BUN: Blood urea nitrogen
CT: Computed tomography
HBI: Fractionated hemibody irradiation
INRG: International Neuroblastoma Risk Group
MIBG: Meta-iodobenzylguanidine
NB: Neuroblastoma
PRA: Plasma renin activity
VMA: Vanillylmandelic acid
